# Correction: Uncovering the Nutritional Landscape of Food

**DOI:** 10.1371/journal.pone.0127128

**Published:** 2015-04-29

**Authors:** The PLOS ONE Staff

## Notice of Republication

This article was republished on April 6, 2015, to correct the captions for Tables 1 and 2, which were mistakenly incorporated into the body text during the production process. The publisher apologizes for the error. Please download this article again to view the correct version. The originally published, uncorrected article and the republished, corrected article are provided here for reference.

## Supporting Information

S1 File Originally published, uncorrected article. (PDF)

S2 File Republished, corrected article. (PDF)

## References

[1] Kim S, Sung J, Foo M, Jin Y-S, Kim P-J (2015) Uncovering the Nutritional Landscape of Food. PLoS ONE 10(3): e0118697 doi: 10.1371/journal.pone.0118697 25768022 10.1371/journal.pone.0118697 PMC4359127

