# Supplementary material for: Uncovering the Nutritional Landscape of Food
Source: PLoS One. 2015 Mar 13;10(3):e0118697. doi: 10.1371/journal.pone.0118697 (PMC4359127; doi:10.1371/journal.pone.0118697)
Supplement: S1 Appendix — (PDF) [file pone.0118697.s001.pdf]

Uncovering the nutritional landscape of food

Seunghyeon Kim, Jaeyun Sung, Mathias Foo, Yong-Su Jin, and Pan-Jun Kim

Appendix S1

Table of Contents

SI Materials and Methods 4

1 Dataset 4

1.1 Nutritional composition and price of food 4

1.2 Recommended levels of nutrient intakes 4

1.3 Consolidation of foods that have almost identical nutrient contents 5

2 Common techniques in this study 6

2.1 Calculation of the Pearson correlation 6

2.2 Calculation of a two-sided *P* value 6

3 The food-food network 6

3.1 Nutritional similarity between foods 6

3.2 Identification of food categories and clusters 7

3.3 Visualization of the food-food network 8

3.4 Case study: northern pike liver and sprouted radish seeds 9

3.5 Case study: finfish and poultry 9

4 Nutritional fitness 10

4.1 Calculation of the nutritional fitness 10

4.2 Composition of an irreducible food set 11

4.3 Nutritional fitness versus price 11

5 Bottleneck nutrients 12

5.1 Identification of bottleneck nutrients 12

5.2 Relation between nutrient availability and favorability 13

5.3 Bottleneck nutrients of different food clusters 13

6 Synergistic bottleneck effects..14
6.1 Characterization of synergistic bottleneck effects................................ 14
6.2 Relation with a correlation between nutrient abundances 15
7 The nutrient-nutrient network 16
7.1 Correlation between nutrient abundances.. 16
7.2 Assembly of the network structure.. 16
7.3 Modular organization of the nutrient-nutrient network 17
7.4 Case studies on robust connections between nutrients 18
SI References 21
SI Figures... 22
SI Tables... 29

SI Materials and Methods

1 Dataset

1.1 Nutritional composition and price of food

We accessed the USDA National Nutrient Database for Standard Reference, Release 24 [1]. The database provides the contents of 7,907 foods in terms of their energy (calorie) and nutrients. The nutrient contents were normalized to the sum (100 g) of protein, total lipid, carbohydrate, water, ash, and alcohol of each food. From these foods, we examined raw foods, as well as other foods whose nutrient contents have been minimally modified. Specifically, we selected foods that fall into one of the following categories: (i) Foods obtained directly from nature or products directly from agriculture, fishery, or livestock farming, without any explicitly added or fortified ingredients such as salt, sugar, and vitamins. Those foods include various raw vegetables, fruits, meat, and fish. (ii) Foods that belong to (i) but have some modifications in their physical properties. Those foods include ground products, e.g., wheat flour and ground meat. (iii) Foods that belong to (i) or (ii) but have additional, minor modifications in their nutrient contents, i.e., frozen, dried, low-fat, and nonfat products. In total, 1,068 foods were selected, and herein, all of them are just called raw foods. For these raw foods, we collected information about their prices from the Center for Nutrition Policy and Promotion (CNPP) Food Prices Database, 2001–02 and 2003–04 [2], [3] and from various online grocery shopping websites.

1.2 Recommended levels of nutrient intakes

For the recommended daily levels of nutrient intakes, we referred to the Dietary Reference Intakes (DRI) published by the Institute of Medicine of the National Academies [4], the Dietary Guidelines for Americans 2010 [5], and the 2002 Joint WHO/FAO Expert Consultation recommendations [6]. We mainly used the data from the first source, while the second and third sources were references only for the data on cholesterol, saturated fatty acids, and *trans*-fatty acids.

The specific values for the lower and upper bounds of the recommended daily intake of nutrients depend on ages and genders. We considered the case of a 20-year-old male, as

shown in Table A. The daily recommended energy E was set to 3057.39 kcal, following the formula E (kcal, for a ≥19-year-old male) = 662 – (9.53 × y) + P_a × (15.91 × w + 539.6 × h), where y (= 20) denotes the age in years, P_a (= 1.27) stands for the physical activity level, w (= 70) is the weight in kg, and h (= 1.77) is the height in m. The reference values of w and h for a 20-year-old male were taken from the DRI.

1.3 Consolidation of foods that have almost identical nutrient contents

We consolidated raw foods that have almost identical nutrient contents by calculating the following quantity for foods i and j:

$$F_{ij} = \min_{K \geq 0} \frac{1}{n_{ij}} \sum_{m=1}^{n_{ij}} \frac{\left(Ka_{im} - a_{jm}\right)^2}{\left(Ka_{im} + a_{jm}\right)^2}, \qquad [1]$$

where $a_{i(j)m}$ is the density of nutrient m in food i (j), n_{ij} is the total number of nutrient m's for foods i and j, and K is a positive real number selected to minimize F_{ij} given $\{a_{im}\}$ and $\{a_{jm}\}$. In Eq. **1**, we only considered nutrient m's (among the 67 nutrients in Table B), which have explicit records of their quantities in both foods i and j and have non-zero quantities for food i or j. The resulting F_{ij} ranges from zero to one, and a small F_{ij} indicates that the foods i and j are similar in their relative nutrient amounts. The calculation of F_{ij} works even with nutrients on very different scales or with different units for the quantities (e.g., μg RAE for vitamin A, and μg DFE for folate). From a probability distribution of F_{ij} over all pairs of foods i and j, we found a sharp transition of the distribution at F_{ij} ~ 0.012. Accordingly, we created unified groups of foods; each group forms an isolated, single connected component in a network of foods linked through F_{ij} < 0.012. For each unified group, the nutrient quantities (per 100 g) and prices were averaged over the foods. The averages for the nutrients were calculated only from foods that had explicit records for the nutrient quantity and were not dried or frozen (differences in the water contents of foods cause large variations in nutrient densities, despite the similar nutrient compositions of the foods). By treating each unified group as a single food, we obtained a total of 654 foods for our analysis (S1 Dataset).

2 Common techniques in this study

2.1 Calculation of the Pearson correlation

The Pearson correlation coefficient (r) between two variables X_i and Y_i (i = 1, 2,⋯, N) is easily distorted by the presence of outliers. When we measured the Pearson correlation in this study, we excluded outliers as follows: $x_i = (X_i - \mu_x)/\sigma_x$ and $y_i = (Y_i - \mu_y)/\sigma_y$, where $\mu_{x(y)}$ and $\sigma_{x(y)}$ are the average and standard deviation of X_i (Y_i), respectively. In a Cartesian plane, we drew a link connecting the data points $P_i = (x_i, y_i)$ and $P_{i'} = (x_{i'}, y_{i'})$ if the Euclidean distance between P_i and $P_{i'}$ was shorter than a certain cutoff d_c (here we chose $d_c = \sqrt{3}$). In this 'network' of data points, we identified the data points in the largest connected component and considered the others to be outliers. The Pearson correlation was measured only for the data points in the largest connected component.

2.2 Calculation of a two-sided *P* value

Let $\{a_i\}$ (i = 1, 2,⋯, N) be a sequence of random numbers in ascending order from a null distribution. Using $\{a_i\}$, we obtain the two-sided P value of a given number X as follows: a value Λ (P =1 if $X = \Lambda$) is expressed as

$$\Lambda = \begin{cases} a_{k+1}, & \text{if } N = 2k+1 \text{ for an integer } k \\ \frac{a_k + a_{k+1}}{2}, & \text{if } N = 2k \text{ for an integer } k \end{cases}, \qquad [2]$$

and the two-sided P value of X is given by

$$P = \begin{cases} \left(\frac{\#\text{ of } a_i \text{ satisfying } a_i \leq X}{\#\text{ of } a_i \text{ satisfying } a_i \leq \Lambda}\right), & \text{if } X \leq \Lambda \\ \left(\frac{\#\text{ of } a_i \text{ satisfying } a_i \geq X}{\#\text{ of } a_i \text{ satisfying } a_i \geq \Lambda}\right), & \text{if } X > \Lambda \end{cases}. \qquad [3]$$

3 The food-food network

3.1 Nutritional similarity between foods

To construct a food-food network that connects foods of similar nutrient contents, we first calculated F_{ij} using Eq. **1** for foods i and j (out of the 654 foods described in Section 1.3). The statistical significance of F_{ij} was assessed based on the following null model: for food i (j), we generated 10,000 random counterparts. For each nutrient in a given counterpart, we

randomly selected a food having an explicit record of the nutrient from all 654 foods, we identified the density of that nutrient in the food, and we assigned the density to the counterpart. These random assignments were only performed for the nutrients that a food *i* (*j*) had explicit records for the quantities. After the counterparts had their respective nutritional compositions, we calculated $F_{i'j'}$ for a pair of counterparts *i'* and *j'* of the foods *i* and *j*, respectively. Using the $F_{i'j'}$ values from 10,000 randomly matched pairs of counterparts, we obtained a *P* value of F_{ij} (Eq. **3**) and the value Λ_{ij} (P =1 if $F_{ij} = \Lambda_{ij}$; Eq. **2**). If $P < 2 \times 10^{-3}$ from Eq. **3**, we extrapolated the *P* value using the estimation $P \propto F_{ij}^{\gamma}$ at $F_{ij} \to 0$. Finally, w_{ij}, the nutritional similarity between foods *i* and *j*, was defined as

$$w_{ij} = \begin{cases} 1 - \dfrac{F_{ij}}{\Lambda_{ij}} & \text{if } F_{ij} < \Lambda_{ij} \\ 0 & \text{otherwise} \end{cases}. \qquad [4]$$

w_{ij} ranges from zero to one, and a large w_{ij} suggests that foods *i* and *j* have similar nutritional compositions. The food-food network consists of foods connected through the w_{ij} weights between foods.

3.2 Identification of food categories and clusters

We conducted an average-linkage hierarchical clustering of the foods (agglomerating foods with high w_{ij} in the food-food network) and built a dendrogram, in which each leaf is a food from the network and branches represent groups of foods (Figure A). Groups that are deeper in the hierarchical levels from the root to leaves contain foods with greater similarity than less deep groups.

In Figure A, near the root, six foods (raw, dried, and frozen egg whites, duck and goose fat, honey, and table salt) are first split from the others because their nutrient contents are dissimilar to those of most foods. The remaining foods are divided into two large parts – animal-derived and plant-derived. The animal-derived part has a layered, core-peripheral organization: the core region (bulky clusters of foods) at the deeper hierarchical level includes protein-rich foods, while the peripheral region outside the core includes both protein-rich and fat-rich foods. In a similar fashion, the plant-derived part is divided into the subcategories shown in Figure A, which are more complex than those in the animal-derived case. The category “protein-rich” encompasses foods having large total amounts of protein. Likewise, “fat-rich” and “carbohydrate-rich” encompass foods having large total amounts of

fat and carbohydrates, respectively. The “low-calorie” category includes foods with relatively low energy (calorie). A fat-rich category is found in both animal-derived and plant-derived foods; however, the animal-derived foods in this category contain much more saturated fatty acids (32.4 ± 9.9 g/100 g) than do the plant-derived foods (9.0 ± 10.7 g/100 g for foods with non-zero amounts of saturated fatty acids).

Each of the above categories contains foods with a wide range of nutrient contents. To identify the most relevant food clusters, it was necessary to determine the best level at which to cut the dendrogram. We chose the threshold that maximizes the number of clusters having ≥4 foods each: this threshold corresponded to $\langle w_{ij} \rangle_{IJ} = 0.7$ (given clusters I and J, $\langle w_{ij} \rangle$ denotes the average w_{ij} over all pairs of food i in cluster I and food j in cluster J). Cutting the dendrogram at this threshold yielded 41 clusters each containing ≥4 foods (S1 Dataset). However, 151 foods did not belong to any of those clusters. Of these, 148 foods were classified into 11 different groups (S1 Dataset) based on the food classes considered by the USDA National Nutrient Database [1]. Because these 11 groups were not from the dendrogram itself, the foods in each group were not necessarily homogeneous in terms of their food categories. The other three foods – duck and goose fat, honey, and salt – were left unclassified.

3.3 Visualization of the food-food network

Because the food-food network was too dense for direct visualization, we extracted a ‘backbone’ structure of the network (Fig. 1); this structure provides informative links that reveal the network’s hierarchical organization. First, we removed six foods of the branches nearest to the root in the dendrogram (Section 3.2). Next, for every branch of the rest of the dendrogram, we identified the two largest sub-trees I and J that bifurcated from the branch into deeper levels of the hierarchy. Among the links between two foods, one food i belonging to sub-tree I and another food j belonging to sub-tree J, we only considered links with $w_{ij} \geq w_{IJ}^{max} - \alpha(w_{IJ}^{max} - \langle w_{ij} \rangle_{IJ})$ for the backbone network. Here, w_{ij} is the nutritional similarity between foods i and j (Eq. **4**), w_{IJ}^{max} and $\langle w_{ij} \rangle_{IJ}$ are the largest and average values of w_{ij} (from sub-trees I and J), respectively, and α is a control parameter ($0 \leq \alpha \leq 1$) to adjust link density of the backbone network. We chose $\alpha = 0.2$ for the network structure presented in Fig. 1.

3.4 Case study: northern pike liver and sprouted radish seeds

In the food-food network, some animal-derived foods are nutritionally similar to plant-derived foods and vice versa. Among them, the livers of northern pikes and sprouted radish seeds have the largest w_{ij} (w_{ij} = 0.68; Eq. **4**). To pinpoint which nutrients of the two foods contribute to their large w_{ij} (or small F_{ij} in Eq. **1**), we identified nutrient m's having small values of $(Ka_{im} - a_{jm})^2/(Ka_{im} + a_{jm})^2$ from Eq. **1**. Among the n_{ij} nutrients, iron has the smallest value of $(Ka_{im} - a_{jm})^2/(Ka_{im} + a_{jm})^2 = 0.0015$, with Ka_{im} = 0.92 mg for northern pike liver and a_{jm} = 0.85 mg for the sprouted radish seeds. Following the ascending order of $(Ka_{im} - a_{jm})^2/(Ka_{im} + a_{jm})^2$, we found that niacin ($Ka_{im}$ = 2.2 mg for northern pike liver, a_{jm} = 2.8 mg for sprouted radish seeds) and fat (Ka_{im} = 3.5 mg for northern pike liver, a_{jm} = 2.5 mg for sprouted radish seeds) are also nutrients for which relative amounts are similar for northern pikes liver and sprouted radish seeds. Throughout this study, we applied the same method when we identified nutrients that two foods have with similar relative amounts.

3.5 Case study: finfish and poultry

The organismal sources of the foods in each food cluster (Section 3.2) were found to be generally homogenous or similar based on their phylogenetic lineage. However, finfish and poultry belonged to the same two clusters. We therefore investigated whether this result implies that finfish and poultry have similar nutrient contents in general. For this purpose, we defined the W_{ij} for foods i and j: if $F_{ij} < \Lambda_{ij}$, $W_{ij} = w_{ij}$, otherwise, $W_{ij} = (\Lambda_{ij} - F_{ij}) / (1 - \Lambda_{ij})$ (for notations, refer to Section 3.1). W_{ij} can be regarded as a natural extension of w_{ij}. We calculated $\delta_{FPO} = \langle W_{ij}\rangle_{FP} - \langle W_{ij}\rangle_{FO}$ and $\delta_{PFO} = \langle W_{ij}\rangle_{FP} - \langle W_{ij}\rangle_{PO}$, where $\langle W_{ij}\rangle_{FP}$ stands for the average W_{ij} of food i in finfish and food j in poultry, and $\langle W_{ij}\rangle_{F(P)O}$ represents the average W_{ij} of food i in finfish (poultry) and food j other than finfish and poultry. It turns out that δ_{FPO} = 0.13 and δ_{PFO} = 0.09. Given the numbers of food pairs between finfish and poultry and between finfish (poultry) and others, we randomly shuffled the W_{ij}'s of the food pairs across the board, and we calculated a P-value of $\delta_{FP(PF)O}$ (Eq. **3**). $P < 2.0\times10^{-5}$ for both δ_{FPO} and δ_{PFO}, indicating that finfish and poultry are significantly similar in their nutrient contents.

4 Nutritional fitness

4.1 Calculation of the nutritional fitness

To calculate the nutritional fitness (NF) of each food, we start by constructing irreducible food sets; each is a set of a small number of different foods. These foods satisfy our daily nutrient demands, and they are not a superset of any other irreducible food set. To obtain a collection of irreducible food sets, we generated an initial food set by solving the following mixed-integer linear programming problem:

$$\text{Minimize } \sum_i q_i \quad [5]$$

Subject to:

$$0 \le x_i \le q_i W \quad [6]$$

$$\sum_i e_i x_i = E \quad [7]$$

$$L_j \le \sum_i a_{ij} x_i \le U_j \quad [8]$$

$$Q_j E \le \sum_j a_{ij} c_j x_i \le R_j E \quad [9]$$

$$\sum_i x_i \le W \quad [10]$$

where q_i is a binary variable (if food i is in the food set, $q_i = 1$; otherwise, $q_i = 0$), x_i is a real variable for the weight of food i to consume per day, E is the daily recommended energy (calorie) (Section 1.2), L_j (U_j) is the lower (upper) bound of the daily recommended intake of nutrient j (Table A), Q_j (R_j) is similar to L_j (U_j) but defined by the % of total energy (Table A), W is the limit of the total weight of daily food consumption ($W = 4$ kg in this study), e_i is the energy density of food i, a_{ij} is the density of nutrient j in food i, and c_j is the energy density of nutrient j.

The solution to Eqs. **5**–**10** gives a food set with four different foods (i.e., $\sum_i q_i = 4$). Next, we expanded the collection of food sets by subsequently adding new food sets to the collection. At each step of adding a new food set, this food set is a solution of Eqs. **5**–**10**, and is constrained to not be a superset of any previous food set in the collection. In this study, we

only considered food sets that each contains less than six different foods (i.e., $\sum_i q_i < 6$). If it was not feasible to find more food sets for the collection, the process was terminated. The final collection comprises all irreducible food sets, 20,476 sets in total. Mathematically, such a collection of irreducible food sets is uniquely determined and has no degeneracy. In this study, the NF_i of food i is given by $NF_i = \log(f_i+1)/\log(N+1)$, where f_i is the number of irreducible food sets including food i, and N is the total number of irreducible food sets. NF_i ranges from zero to one, and a large NF_i indicates that food i is nutritionally favorable. For the generalized definition of NF_i, any functional form that monotonically increases with f_i is acceptable, as long as only ordinal information of NF_i matters. Note that f_i is capable of quantifying NF_i under the condition of small $\sum_i q_i$ as in this study. Otherwise, it may be hard to estimate the true nutritional adequacy of food i' using solely $f_{i'}$. For example, a nutritionally poor food i' in an irreducible food set will be easily complemented by many other foods (in the same set) to satisfy Eqs. **7**–**10** if $\sum_i q_i$ is not small enough.

4.2 Composition of an irreducible food set

As mentioned in Section 4.1, the minimum size of an irreducible food set is 4. There are 34 such irreducible food sets. These food sets are composed of foods covering all of the four major food categories described in Section 3.2. For example, one of these irreducible food sets includes northern pike (x_i = 0.7 kg) in the protein-rich category, almond (x_i = 0.2 kg) in the fat-rich category, cherimoya (x_i = 1.8 kg) in the carbohydrate-rich category, and Swiss chard (x_i = 0.5 kg) in the low-calorie category. Another includes flatfish (x_i = 0.6 kg) in the protein-rich category, almond (x_i = 0.2 kg) in the fat-rich category, cherimoya (x_i = 1.3 kg) in the carbohydrate-rich category, and red cabbage (x_i = 1.9 kg) in the low-calorie category. In the cases of irreducible food sets having five foods each (20,442 food sets in total), the majority of the food sets (93.7%) were also composed of foods covering all four major food categories.

4.3 Nutritional fitness versus price

We investigated whether there is any correlation between the NF of a food and its price per weight (Section 1.1). Fig. 2C and Figure E show essentially no correlation between these factors. In this analysis, we excluded two foods with extremely high prices to prevent any misleading results: one was saffron (3,607.9 USD/100 g) and the other was sea cucumber

(yane, Alaska native, 105.7 USD/100 g).

5 Bottleneck nutrients

5.1 Identification of bottleneck nutrients

In each food category, we identified bottleneck nutrients for high NF as follows. For nutrient i and food j, we calculated $M_{ij}^{L} = \langle n_{jk}^{iL}/N_{jk}\rangle_k$. Here, N_{jk} is the number of irreducible food sets that include food j and follows Eq. **10** when food k substitutes for food j using x_k that satisfies Eq. **7**. Among N_{jk} irreducible food sets, n_{jk}^{iL} denotes the number of sets that no longer satisfy the inequalities in Eqs. **8** and **9** for the lower bounds of nutrient i when food k substitutes food j using x_k that satisfies Eq. **7**. Food j is selected from the top-20%-NF foods in a given food category (an exception is the fat-rich category, in which we consider the top-5-NF foods that have a distinctively higher NF than the others), and food k is selected in the same category from the foods that are not the top NF foods. $\langle\cdots\rangle_k$ denotes the average over the food k's. Likewise, we also calculated $M_{ij}^{U} = \langle n_{jk}^{iU}/N_{jk}\rangle_k$, where n_{jk}^{iU} is the number of irreducible food sets that no longer satisfy the inequalities in Eqs. **8** and **9** for the upper bounds of nutrient i when food j is replaced with food k. For nutrient i, if there exists at least one food j with $M_{ij}^{L(U)} > 0.5$, we place the nutrient i in a tentative list of bottleneck nutrients that may be favorable (unfavorable) for high NF in the food category of food j. In this tentative list of bottleneck nutrients, there was no nutrient that can be both favorable and unfavorable for high NF.

Given a food category, we performed a linear regression for the NF of a food in that category using the tentative bottleneck nutrients in the food:

$$\mathrm{NF}_n = \sum_i \alpha_i \frac{A_{ni}}{\langle A_{mi}\rangle_m} + \beta + \epsilon_n\,, \qquad [11]$$

where NF_n is the NF of food n, α_i is a regression coefficient of tentative bottleneck nutrient i, A_{ni} is the density of nutrient i in the dry matter of food n, $\langle A_{mi}\rangle_m$ is an average of A_{mi} over the food m's having $A_{mi} > 0$, β denotes a constant specific to the food category, and ε_n denotes the regression error. If the regression in Eq. **11** gives rise to $\alpha_i \leq 0.01$ ($\alpha_i \geq -0.01$) for some tentatively favorable (unfavorable) nutrients, we exclude the nutrients from Eq. **11** and repeat the regressions until no such α_i's exist. If the resultant α_i is > 0.01 (α_i is < -0.01) for a

tentatively favorable (unfavorable) nutrient *i*, the nutrient is finally considered to be a bottleneck nutrient for high NF in that food category.

5.2 Relation between nutrient availability and favorability

For each food *j* in a given food category, we consider a certain amount of food *j*, X_j, where $X_j = \min(E/e_j, W)$ (see Section 4.1 for notations here). Using $\{X_j\}$, we obtain V_i^L for each nutrient *i*; V_i^L is defined as the fraction of food *j*'s (in the food category) satisfying $a_{ji}X_j < L_i$ or $a_{ji}c_iX_j < Q_iE$. In a similar manner, we obtain V_i^U, which is defined as the fraction of food *j*'s (in the food category) satisfying $U_i < a_{ji}X_j$ or $R_iE < a_{ji}c_iX_j$. In other words, $V_i^{L(U)}$ roughly quantifies how scarce (excessive) nutrient *i* is in a food of a given food category. Figure F shows that favorable bottleneck nutrients tend to have a higher V_i^L than that of the other nutrients, whereas unfavorable bottleneck nutrients tend to have a higher V_i^U than that of the other nutrients.

5.3 Bottleneck nutrients of different food clusters

In Fig. 2D and Figure C, foods from different clusters of the same category have moderately distinguishable NFs. In a manner analogous to Section 5.1, we identified which nutrients to contribute to the NF differences between clusters. For a cluster with relatively high NFs in a particular food category, we first identify the food *j*'s (in that cluster) having similar NFs to the cluster average. For such food *j* and nutrient *i*, we calculate M_{ij}^L and M_{ij}^U, which are analogous to those in Section 5.1. For this calculation, food *j* in the irreducible food sets is replaced by foods with NFs lower than the NF of food *j*. Those foods are selected from clusters (and food groups not belonging to any clusters, see Section 3.2) having average NFs lower than the average NF for food *j*'s cluster. If $M_{ij}^{L(U)} > 0.5$, we can consider that nutrient *i* is not sufficiently found (much abundantly found) in other types of foods, and thus, nutrient *i* contributes to the high NFs of the food cluster containing food *j*. A similar idea can be applied to a cluster with relatively low NFs. In this case, food *j* in the low-NF cluster is used to replace the foods of higher NFs in irreducible food sets, to identify which nutrients are problematic in this low-NF cluster.

In the protein-rich category, finfish and animal liver clusters tend to have high NFs. Finfish clusters have sufficient amounts of choline and vitamin D, and a cluster of animal livers has sufficient amounts of vitamin A. Among the low-NF clusters, the beef cluster has insufficient

amounts of choline and excessive amounts of niacin, and poultry has insufficient amounts of choline. In fact, choline, vitamin D, and niacin are all bottleneck nutrients in the protein-rich category identified in Section 5.1. Finfish of the ‘Finfish (with some shellfish and poultry)’ cluster contains 49.5 ± 27.1 mg/100 g of choline, while poultry of the ‘Poultry (with some beef and lamb)’ cluster contains only 6.2 ± 12.4 mg/100 g of choline.

In the fat-rich category, the seed and nut clusters tend to have high NFs, with sufficient amounts of choline. An animal fat cluster has low NFs with insufficient amounts of choline and excessive amounts of cholesterol and saturated fatty acids. The ‘Seeds (mixed1)’ cluster contains 31.9 ± 31.0 mg/100 g of choline and 4.4 ± 1.3 g/100 g of saturated fatty acids without any cholesterol. In contrast, the ‘Animal fat and suet’ cluster contains 1.4 ± 2.4 mg/100 g of choline, 79.6 ± 8.9 mg/100 g of cholesterol, and 38.3 ± 8.3 g/100 g of saturated fatty acids.

In the carbohydrate-rich category, the low-NF clusters tend to have excessive amounts of manganese and folate. The ‘Fruits (mixed1)’ cluster, which is a high-NF cluster, contains 0.1 ± 0.1 mg/100 g of manganese and 14.7 ± 7.4 μg DFE/100 g of folate. In contrast,, the ‘Cereal grains’ cluster, which is a low-NF cluster, contains 2.6 ± 2.9 mg/100 g of manganese and 66.4 ± 103.2 μg DFE/100 g of folate.

In the low-calorie category, rhubarb tends to have a high NF with sufficient amount of choline and vitamin K. Peppers have sufficient amounts of vitamin A. Among the low-NF clusters, herbs do not have sufficient amounts of choline, and seed spices do not have sufficient amounts of vitamin A and choline. The ‘Spices (peppers)’ cluster contains 1693.5 ± 480.1 μg RAE/100 g of vitamin A, and the ‘Spices (seeds)’ cluster contains 18.5 ± 21.2 μg RAE/100 g of vitamin A.

6 Synergistic bottleneck effects

6.1 Characterization of synergistic bottleneck effects

Following steps similar to those in Section 5.1, we identified nutrient pairs having synergistic bottleneck effects in NF. For a pair of nutrients *i* and *j* and food *k* in a given food category, we define $\varepsilon_i^k = M_{ik}^L + M_{ik}^U$, $\varepsilon_j^k = M_{jk}^L + M_{jk}^U$, and $\varepsilon_{ij}^k = \langle n_{km}^{ij} / N_{km} \rangle_m$. Here, N_{km} is the number of irreducible food sets. Each includes food *k*, and still satisfies Eq. **10** when we substitute food

m for food k using x_m that satisfies Eq. **7**. Among those N_{km} irreducible food sets, n_{km}^{ij} denotes the number of sets that no longer satisfy Eqs. **8** and **9** for either nutrient i or j when food k is replaced by food m, with x_m satisfying Eq. **7**. Food k is selected from the top-20%-NF foods in a given food category (an exception is the fat-rich category, in which we consider the top-5-NF foods that show a distinctively higher NF than the others), and food m is selected in the same category from those foods not in the top NF foods. $\langle \cdots \rangle_m$ denotes the average over the food m's. For the other notations here, refer to Section 5.1. Accordingly, $\varepsilon_{i(j)}{}^k$ corresponds to the fraction of irreducible food sets (with food k) that no longer satisfy the recommended level of nutrient i (j) when food k is replaced by a food other than the high-NF foods. $\varepsilon_{ij}{}^k$ works similarly, but for a pair of nutrients i and j. Given $\varepsilon_i{}^k$ and $\varepsilon_j{}^k$, we obtained the null distribution of $\varepsilon_{ij}{}^k$ using the assumption that the nutrients i and j are independently distributed over foods. The null distribution can be approximated by a normal distribution with a mean $\varepsilon_i{}^k + \varepsilon_j{}^k - \varepsilon_i{}^k \varepsilon_j{}^k$ and variance $\varepsilon_i{}^k \varepsilon_j{}^k(1-\varepsilon_i{}^k)(1-\varepsilon_j{}^k)/\langle N_{km}\rangle_m$. Finally, $\Phi_{ij}{}^k$ is defined as the Z score of the actual $\varepsilon_{ij}{}^k$ based on this null distribution. $\Phi_{ij}{}^k$ measures the degree of synergism between two nutrients i and j for high NF in food k. We assign $\Phi_{ij}{}^k$ values only in cases where $\varepsilon_{ij}{}^k > 0.5$, i.e., the 'consequence' of the synergism for high NF is strong enough. Although nutrient i or j itself can be a non-bottleneck nutrient (according to Section 5.1), it is called a favorable nutrient if $M_{i(j)k}{}^L/\varepsilon_{i(j)}{}^k \geq 0.8$. If $M_{i(j)k}{}^U/\varepsilon_{i(j)}{}^k \geq 0.8$, it is called an unfavorable nutrient. Given nutrients i and j and a food category, we also use $\Phi_{ij} = \max_k \Phi_{ij}{}^k$ as a representative value of $\Phi_{ij}{}^k$ for the high-NF food k's in the food category.

6.2 Relation with a correlation between nutrient abundances

Given a food category, we collected all pairs of nutrients that satisfy the following conditions: (i) one nutrient is favorable and the other is unfavorable, and (ii) they have Φ_{ij} assigned in Section 6.1. For such pairs of nutrients i and j, we calculated the Pearson correlation r_{ij} (Section 2.1) between the densities of the nutrients i and j across the foods in the category. Here, the density of a nutrient in food was measured as the quantity per dry weight. For nutrients i and j, only foods having explicit records of both nutrient quantities (and at least one nutrient with a non-zero quantity) were considered. If the number of such foods was ≥ 10 after excluding outlier foods (Section 2.1), we measured r_{ij}; otherwise, we omitted the pair of nutrients i and j from our analysis. Fig. 3 shows that highly synergistic nutrient pairs (Φ_{ij} >

2.0) tend to have more positive correlations ($r_{ij} > 0$) than the others ($\Phi_{ij} \leq 2.0$). For each food category, we generated a null model by randomly shuffling the r_{ij}'s of the nutrient pairs, and we calculated the *P*-value of the difference between the average of the actual r_{ij}'s from the pairs with $\Phi_{ij} > 2.0$ and that from the pairs with $\Phi_{ij} \leq 2.0$. Those *P*-values are presented in Fig. 3.

7 The nutrient-nutrient network

7.1 Correlation between nutrient abundances

To construct a nutrient-nutrient network that connects nutrients through correlations of their abundances, we calculated the Pearson correlation r_{ij} between the densities of nutrients *i* and *j* across foods. With a method similar to that in Section 6.2, the nutrient density was measured as the quantity per dry weight. For nutrients *i* and *j*, only foods having explicit records of both nutrient quantities (and at least one nutrient with a non-zero quantity) were considered. If the number of such foods was ≥ 10 after excluding outliers (Section 2.1), we computed r_{ij}; otherwise, we omitted the connection between nutrients *i* and *j* from the network structure.

7.2 Assembly of the network structure

Fig. 4 shows the nutrient-nutrient network based on the correlations across all foods (we also considered the correlations measured in a food-group-specific manner for subsequent analyses). This network comprises only the following significant nutrients and correlations. Among all nutrients, we excluded compounds that appeared in merely ≤ 10 foods and minor fatty acids. Redundant nutrients (e.g., folic acid, food folate, and total folate) were represented by one of those nutrients. We excluded amino acids because their correlations with other nutrients were very similar to the correlations of protein with others, and thus protein could represent all the amino acids. Table E shows the resulting list of such nutrients. Among the pairs of nutrients in Table E, we considered those having significant links for $|r_{ij}| \geq 0.4$ (Section 7.1) and with the false discovery rate controlled at 2.5×10^{-3} ($P \leq 9.8\times10^{-4}$). Seven nutrients do not appear in the network because they have no such significant links.

The statistical significance of links, mentioned above, was measured as follows: for a given pair of nutrients *i* and *j*, we first removed the outlier foods defined in Section 2.1 before

generating the null model. (i) If $r_{ij} > 0$, we selected only the foods having explicit records for both nutrient quantities (and at least one nutrient with a non-zero quantity), and we randomly shuffled the densities (quantity per dry weight) of either nutrient *i* or *j* across those foods. The Pearson correlations between such nutrient densities across the foods constitute the null distribution that gives a *P*-value of r_{ij} (Section 2.2). (ii) If $r_{ij} < 0$, we selected only the foods having explicit records for both nutrient quantities, and we randomly shuffled densities (quantity per dry weight) of either nutrient *i* or *j* across those foods. The Pearson correlation between such nutrient densities was measured only for the foods having at least one nutrient with a non-zero density after shuffling. These Pearson correlations constitute the null distribution that gives a *P*-value of r_{ij} (Section 2.2). The difference between (i) and (ii) provides a stringent test of the significance, depending on the signs of the r_{ij}'s. If $P < 2 \times 10^{-3}$, we extrapolated the *P* value using the estimation $P \propto (1-|r_{ij}|)^{\gamma}$ at $r_{ij} \to \pm 1$ (γ depends on the sign of r_{ij}).

7.3 Modular organization of the nutrient-nutrient network

Fig. 4 shows that the nutrient-nutrient network can be divided into three major groups of nutrients that are densely connected to each other through positive correlations. Between the groups, the nutrients only have sparsely positive or frequently negative correlations. The first group comprises protein, lipids, and a number of micronutrients: specifically, they are protein, cholesterol, riboflavin, pantothenic acid, vitamin B12, niacin, vitamin B6, choline, sodium, phosphorus, selenium, zinc, betaine, menaquinone-4, *trans*-fatty acids, saturated fatty acids, linoleic acid, polyunsaturated fat, monounsaturated fat, and total lipid. The second group contains digestible carbohydrates: carbohydrate, starch, sugar, sucrose, glucose, and fructose. The third group consists of fiber, α-linolenic acid, vitamin A, magnesium, folate, vitamin K, iron, vitamin E, vitamin C, potassium, calcium, and manganese. Although most of the positive correlations occur between nutrients in the same groups, the following two pairs of nutrients from different groups have positive correlations: riboflavin and potassium, and fructose and α-linolenic acid. Although most of the negative correlations occur between nutrients of different groups, the following four pairs of nutrients have intra-group, negative correlations: choline and linoleic acid, starch and sugar, starch and glucose, and starch and fructose.

Each of the three nutrient groups largely captures the nutrient characteristics of a particular food partition or category. For food i and a nutrient group J (J = 1, 2, 3), let $\Omega_{iJ} = \langle A_{ij}/\langle A_{nj}\rangle_n\rangle_{j\in J}$, where A_{ij} is the density of nutrient j in food i's dry matter, $\langle A_{nj}\rangle_n$ is the average of A_{nj} over the food n's having $A_{nj} > 0$, and $\langle\cdots\rangle_{j\in J}$ represents the average over the nutrient j's belonging to the nutrient group J. Thus, Ω_{iJ} quantifies the enrichment of food i's nutrients in a nutrient group J. Figure G clearly reveals that animal-derived foods tend to have high Ω_{iJ}'s in the first nutrient group, while plant-derived, low-calorie foods have high Ω_{iJ}'s in the third group. The fat- and protein-rich foods within the plant-derived food partition tend to have high Ω_{iJ}'s in both the first and third nutrient groups. Carbohydrate-rich foods tend to have high Ω_{iJ}'s in the second and third nutrient groups. One may suppose that these results can be readily expected from the definitions of the food categories themselves, e.g., carbohydrate-rich foods, by definition, contain large proportions of total carbohydrates. However, Figure G shows that our results remained valid after controlling for such trivial factors, i.e., the exclusion of the "Protein", "Total lipid", and "Carbohydrate" nutrient nodes (Fig. 4) from Ω_{iJ} calculations. Furthermore, a stricter calculation of Ω_{iJ} – even without any subcompounds of macronutrients – did not notably change our main results (in this case, the second nutrient group does not have any nutrients for this calculation, so the analysis was performed only for the first and third nutrient groups).

7.4 Case studies on robust connections between nutrients

In the nutrient-nutrient network, protein and choline share a positive correlation across all foods (r = 0.77, $P = 4.0\times10^{-30}$). They also have positive correlations for plant-derived foods (r = 0.52, $P = 9.3\times10^{-13}$) and animal-derived foods (r = 0.71, $P = 5.2\times10^{-11}$) separately. In addition, each subgroup of the animal-derived foods offers positive correlations between protein and choline: r = 0.49 ($P = 3.6\times10^{-4}$) for foods from aquatic animals, r = 0.78 (P = 0.002) for beef, r = 0.94 ($P = 1.4\times10^{-7}$) for pork, and r = 0.95 ($P = 8.7\times10^{-5}$) for chicken. One may raise the possibility that such correlations can be made by indirect relationships resulting from transitivity effects: if nutrients i and j are each correlated with a third common nutrient, nutrients i and j can correlate with each other despite a lack of direct association. We found that, the connection between protein and choline remains valid, even when we

removed such indirect causes of the correlation. For example, phosphorus positively correlates both with protein (r = 0.73, $P = 1.5\times10^{-41}$) and choline (r = 0.72, $P = 2.9\times10^{-17}$). To reduce the indirect correlation made by phosphorus, we measured the correlation between protein and choline only across foods having a particular level of phosphorus. This process controlled for the abundance of phosphorus. Our analysis reveals that protein and choline positively correlate regardless of the controlled levels of phosphorus. Similar results were observed when we controlled for the indirect effects of cholesterol, niacin, vitamin B12, selenium, and zinc, which positively correlate with both protein and choline. All these results consistently support the robust association between protein and choline.

In the nutrient-nutrient network, another example of positive correlations between nutrients can be found between protein and niacin. They exhibit a highly positive correlation across all foods (r = 0.59, $P = 6.3\times10^{-26}$) but also separately for plant-derived foods (r = 0.32, $P = 2.7\times10^{-6}$) and animal-derived foods (r = 0.44, $P = 1.2\times10^{-9}$). In addition, each subgroup within the animal-derived foods offers positive correlations between protein and niacin: r = 0.56 ($P = 5.1\times10^{-4}$) for beef, r = 0.69 ($P = 4.3\times10^{-6}$) for pork, r = 0.67 (P = 0.002) for lamb, and r = 0.78 ($P = 3.5\times10^{-4}$) for chicken. Tryptophan can be converted to niacin in animal livers, and this conversion may contribute, at least in part, to the robust connection between protein and niacin.

In the nutrient-nutrient network, *trans*-fatty acids exhibit a significantly positive correlation with zinc across all foods (r = 0.62, $P = 9.1\times10^{-9}$) and separately for plant-derived foods (r = 0.35, P = 0.05) and animal-derived foods (r = 0.60, $P = 7.8\times10^{-6}$). Because *trans*-fatty acids also share a positive correlation with saturated fatty acids (r = 0.59, $P = 1.4\times10^{-6}$), we considered the possibility that zinc and *trans*-fatty acids may be indirectly correlated through saturated fatty acids. To remove any indirect effects from saturated fatty acids, we measured the correlation between zinc and *trans*-fatty acids only across foods having a particular level of saturated fatty acids. This process controlled for the abundance of saturated fatty acids. Our analysis reveals that zinc and *trans*-fatty acids are still positively correlated to each other as long as the controlled level of saturated fatty acids in food is ≥ 5.8 g/100 g (dry weight). In the nutrient-nutrient network, betaine has a positive correlation both with zinc and *trans*-fatty acids, raising the possibility of indirect effects leading to the correlation between zinc and *trans*-fatty acids. Applying a method similar to that for the case of saturated fatty

acids, we found that zinc and *trans*-fatty acids are still positively correlated as long as the controlled level of betaine in food is ≥ 41.0 μg/100 g (dry weight). Lastly, menaquinone-4 in the network has a strong correlation both with *trans*-fatty acids (r = 0.98, $P = 8.6\times10^{-9}$) and zinc (r = 0.63, $P = 6.4\times10^{-5}$). We suppose that the correlation between menaquinone-4 and *trans*-fatty acids may be analogous to the relationship between 2´,3´-dihydrophylloquinone and *trans*-fatty acids [7] because menaquinone-4 and 2´,3´-dihydrophylloquinone belong to the same vitamin family. Despite the possibility of indirect effects from menaquinone-4, our analysis shows that *trans*-fatty acids and zinc positively correlate with each other even among foods that lack menaquinone-4.

SI References

1. U.S. Department of Agriculture, Agricultural Research Service (2011) USDA National Nutrient Database for Standard Reference, Release 24. Nutrient Data Laboratory Online. Available: http://www.ars.usda.gov/Services/docs.htm?docid=22808. Accessed 31 December 2011.
2. U.S. Department of Agriculture, Center for Nutrition Policy and Promotion (2008) CNPP Food Prices Database, 2001-02. Available: http://www.cnpp.usda.gov/USDAFoodPlansCostofFood. Accessed 1 March 2012.
3. U.S. Department of Agriculture, Center for Nutrition Policy and Promotion (2009) CNPP Food Prices Database, 2003-04. Available: http://www.cnpp.usda.gov/USDAFoodPlansCostofFood. Accessed 1 March 2012.
4. National Research Council (2005) Dietary Reference Intakes for Energy, Carbohydrate, Fiber, Fat, Fatty Acids, Cholesterol, Protein, and Amino Acids (Macronutrients). Washington, DC: The National Academies Press.
5. U.S. Department of Agriculture, U.S. Department of Health and Human Services (2010) Dietary Guidelines for Americans, 2010, 7th Ed. Washington, DC: U.S. Government Printing Office.
6. Nishida C, Uauy R, Kumanyika S, Shetty P (2004) The Joint WHO/FAO Expert Consultation on diet, nutrition and the prevention of chronic diseases: process, product and policy implications. Public Health Nutr 7: 245–250.
7. Booth SL (2012) Vitamin K: food composition and dietary intakes. Food Nutr Res 56: 5505.

SI Figures

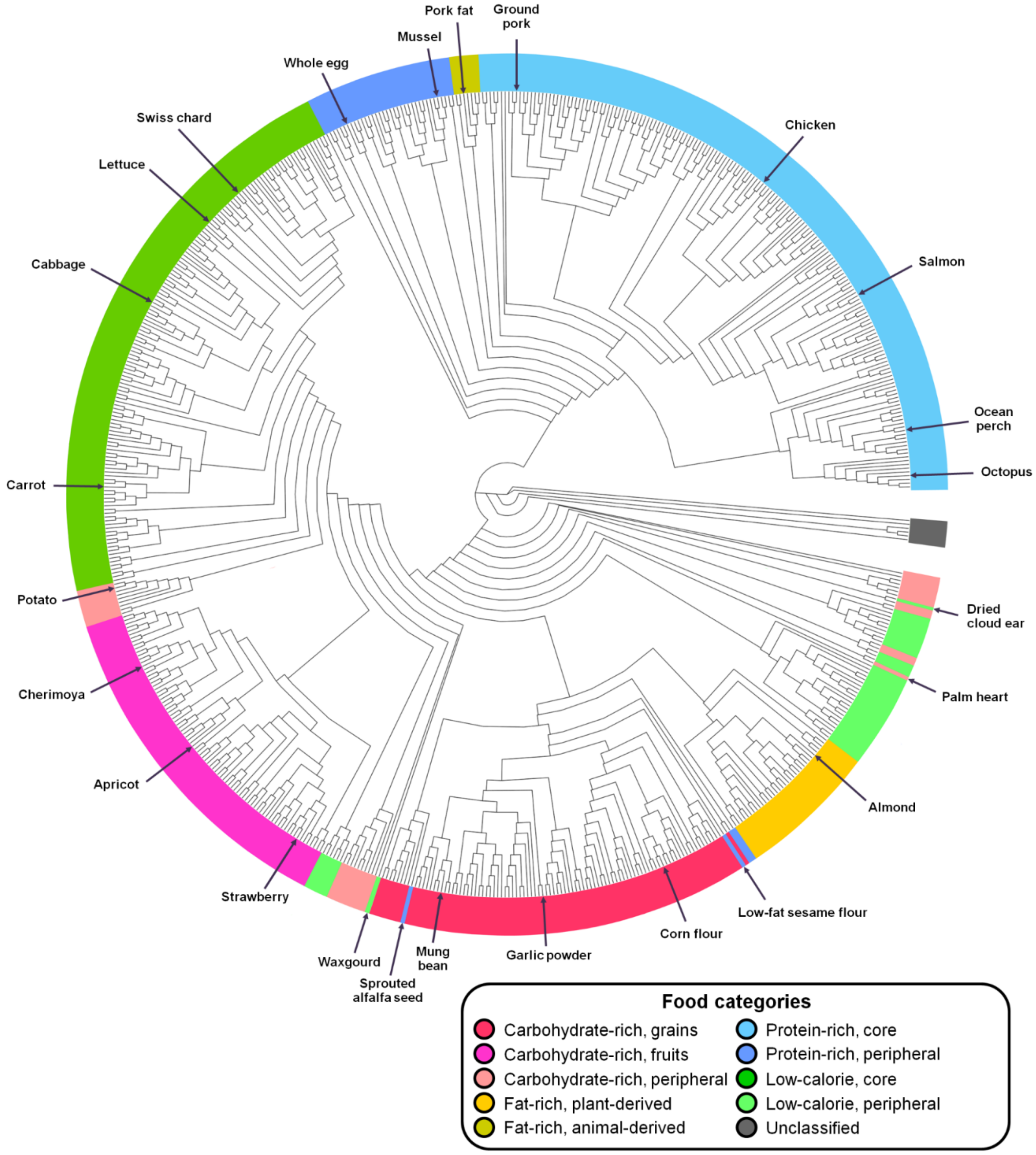

Figure A. Dendrogram of 654 foods based on the nutritional similarity between foods (Section 3.2). Terminal nodes correspond to different foods. According to a hierarchical organization of nutritionally similar foods and their macronutrient (or energy) densities, we

classified the foods into several subcategories, as denoted by the colored bands. The tree structure only represents a hierarchical organization of foods, and the branch lengths do not quantitatively represent nutritional similarities.

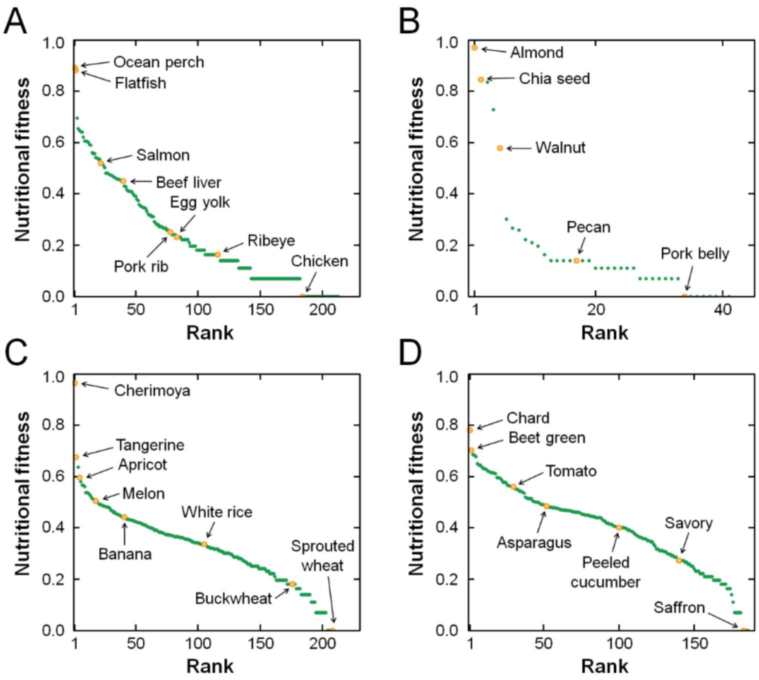

Figure B. Nutritional fitness (NFs) of foods (sorted in descending order) from each food category. (A) Protein-rich category. (B) Fat-rich category. (C) Carbohydrate-rich category. (D) Low-calorie category.

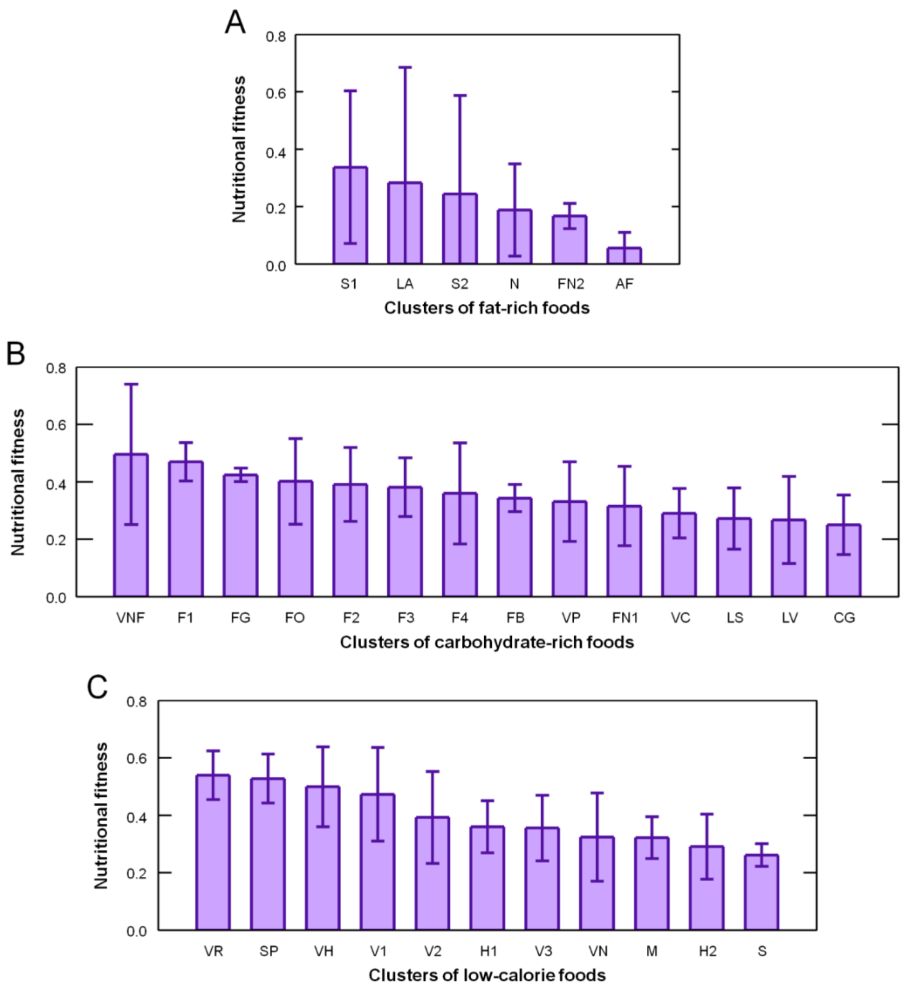

Figure C. NFs of foods (average and standard deviation) in each cluster of a given food category. The result for the protein-rich category is shown in Fig. 2D. (A) Fat-rich category. Clusters are abbreviated as follows. S1: Seeds (mixed1); LA: Legumes (with almond); S2: Seeds (mixed2); N: Nuts (mixed); FN2: Fruits and nuts (mixed2); AF: Animal fat and suet. (B) Carbohydrate-rich category. Clusters are abbreviated as follows. VNF: Vegetables, nuts, and fruits; F1: Fruits (mixed1); FG: Fruits (dried grapes); FO: Fruits (mostly oranges and grapefruits); F2: Fruits (mixed2); F3: Fruits (mixed3); F4: Fruits (mixed4); FB: Fruits (berries); VP: Vegetables (potatoes); FN1: Fruits and nuts (mixed1); VC: Vegetables (corns); LS: Legumes and spices; LV: Legumes and vegetables; CG: Cereal grains. (C) Low-calorie category. Clusters are abbreviated as follows. VR: Vegetables (rhubarbs and celeries); SP: Spices (peppers); VH: Vegetables and herbs; V1: Vegetables (mixed1); V2: Vegetables (mixed2); H1: Herbs (mixed1); V3: Vegetables (mixed3); VN: Vegetables and nuts; M: Mushrooms; H2: Herbs (mixed2); S: Spices (seeds).

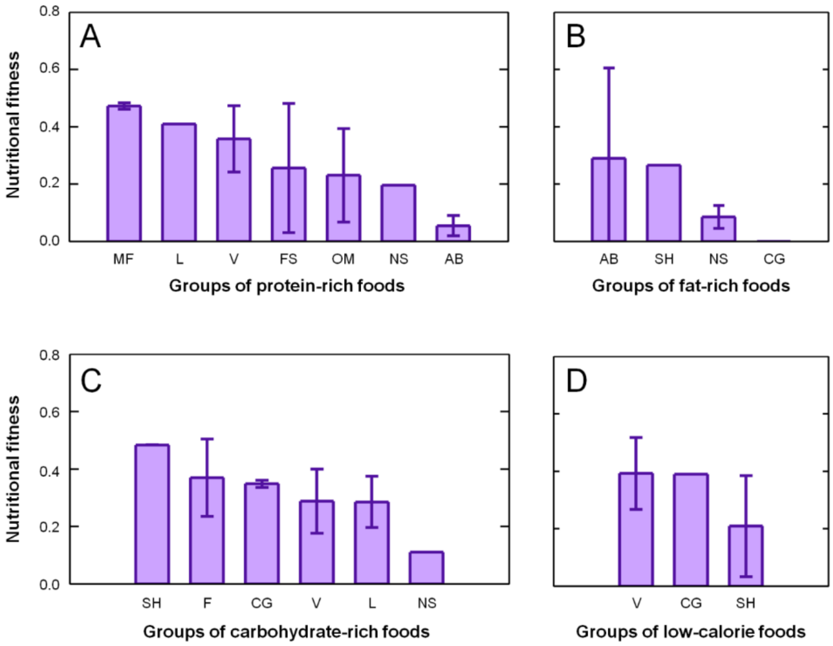

Figure D. NFs of foods (average and standard deviation) in each group of a given food category. The food groups consist of foods not belonging to any food clusters as determined by the dendrogram in Figure A (Section 3.2); thus, the foods in each group are not necessarily homogeneous in terms of their food categories. (A) Protein-rich category. (B) Fat-rich category. (C) Carbohydrate-rich category. (D) Low-calorie category. In (A–D), food groups are abbreviated as follows. MF: Milk (nonfat); L: Legumes; V: Vegetables; FS: Finfish and shellfish; OM: Other meats; NS: Nuts and seeds; AB: Animal byproducts; SH: Spices and herbs; CG: Cereal grains; F: Fruits. We excluded the group “Eggs (white)” as it does not belong to any major food category (Section 3.2).

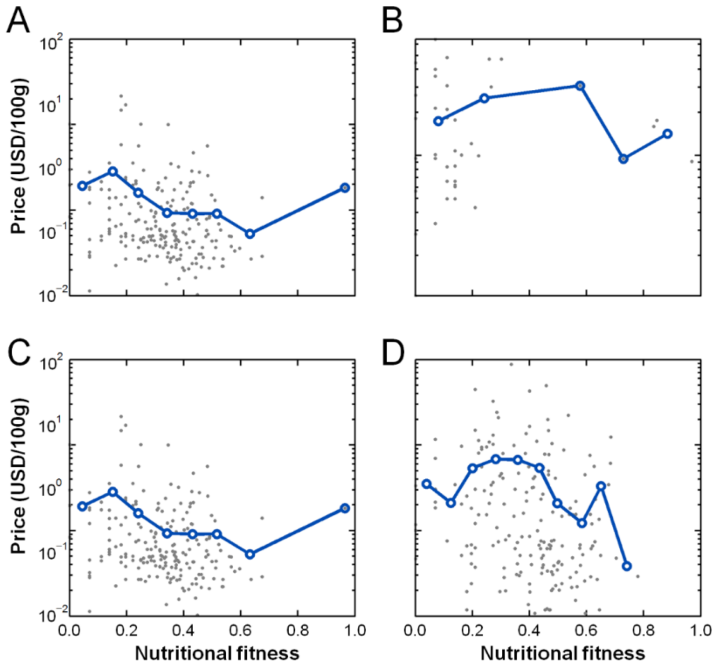

Figure E. NF versus price (per weight) for each food (gray) in a given food category. The blue lines indicate the average prices along the NFs. (A) Protein-rich category (r = 0.02, P = 0.80). (B) Fat-rich category (r = −0.03, P = 0.95). (C) Carbohydrate-rich category (r = −0.20, P = 0.004). (D) Low-calorie category (r = −0.12, P = 0.11). In (A) and (D), we omit two foods with extremely high prices to prevent any misleading results (Section 4.3).

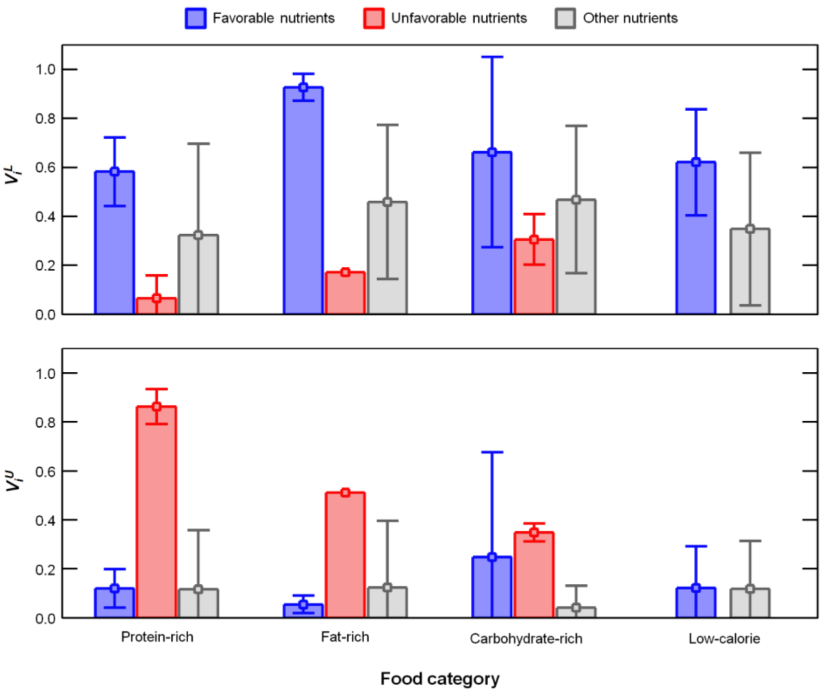

Figure F. Relation between nutrient availability and favorability. V_i^L and V_i^U (averages and standard deviations) are shown for the favorable and unfavorable bottleneck nutrients and the other nutrients (from Table A) in each food category. $V_i^{L(U)}$ measures how scarce (excessive) a nutrient is in food. For details, see Section 5.2.

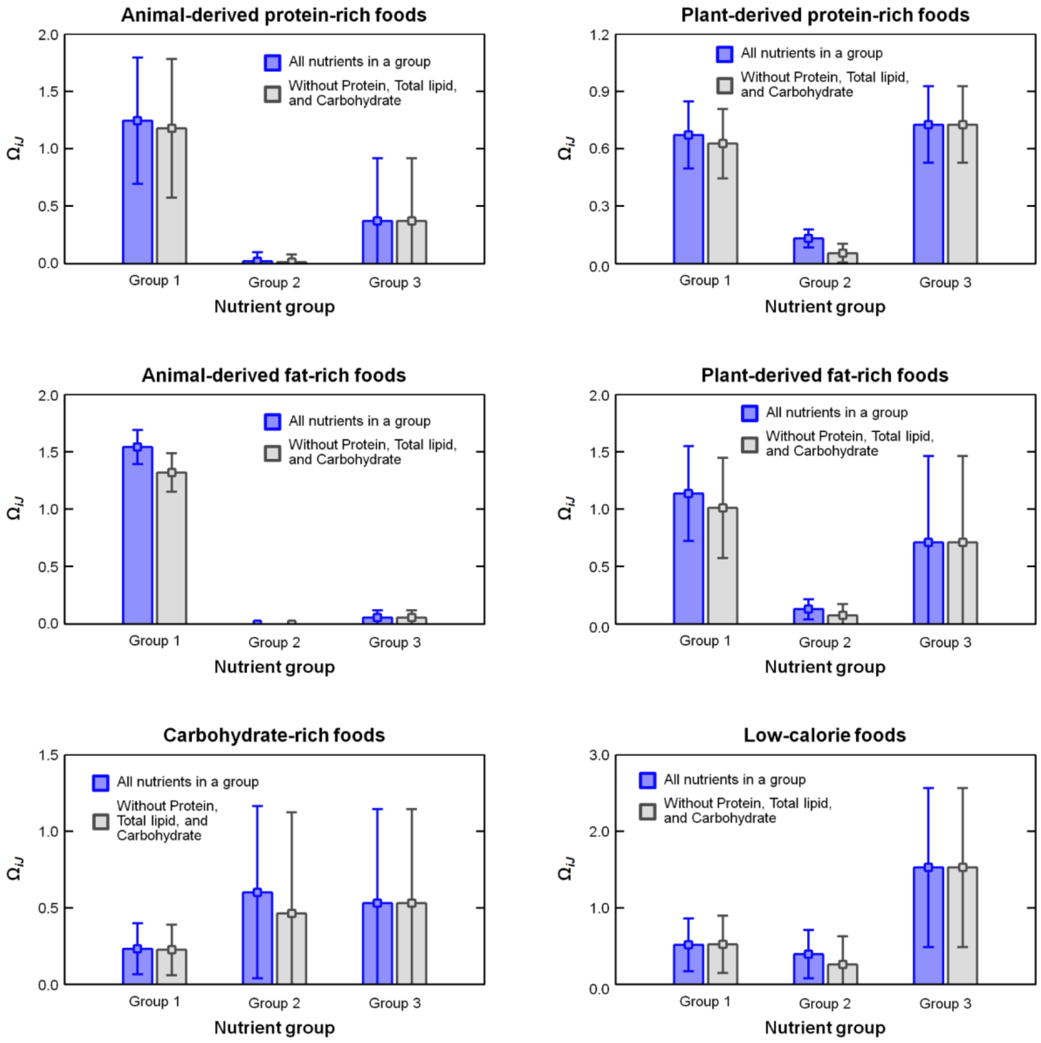

Figure G. Nutrient groups in the nutrient-nutrient network; these groups underlie the nutritional compositions of the different food partitions or categories. Ω_{iJ} represents the enrichment of food's nutrients in a given group of nutrients (Groups 1, 2, 3). The averages and standard deviations of Ω_{iJ}'s are shown for different food partitions or categories. Group 1 includes the components of protein and lipids, and the relevant micronutrients. Group 2 includes digestible carbohydrates. Group 3 includes fiber, α-linolenic acid, and the relevant micronutrients. The Ω_{iJ}'s were calculated for all nutrients or nutrients other than "Protein", "Total lipid", and "Carbohydrate" in Fig. 4. For details, see Section 7.3.

SI Tables

Table A. Daily recommended nutrient intakes for a physically active 20-year-old male

Nutrient ID	Nutrient name	Minimum intake	Maximum intake	Minimum intake (% of calories)	Maximum intake (% of calories)
203	Protein	56 g	ND*	10	35
204	Total lipid	0 g	ND	20	35
205	Carbohydrate	130 g	ND	45	65
291	Fiber	38 g	ND	ND	ND
301	Calcium	1000 mg	2500 mg	ND	ND
303	Iron	8 mg	45 mg	ND	ND
304	Magnesium	400 mg	ND	ND	ND
305	Phosphorus	700 mg	4000 mg	ND	ND
306	Potassium	4700 mg	ND	ND	ND
307	Sodium	1500 mg	2300 mg	ND	ND
309	Zinc	11 mg	40 mg	ND	ND
312	Copper	0.9 mg	10 mg	ND	ND
315	Manganese	2.3 mg	11 mg	ND	ND
317	Selenium	55 µg	400 µg	ND	ND
320	Vitamin A	900 µg RAE†	3000 µg RAE	ND	ND
323	Vitamin E	15 mg	1000 mg	ND	ND
328	Vitamin D	5 µg	50 µg	ND	ND
401	Vitamin C	90 mg	2000 mg	ND	ND
404	Thiamin	1.2 mg	ND	ND	ND
405	Riboflavin	1.3 mg	ND	ND	ND
406	Niacin	16 mg NE‡	35 mg	ND	ND
410	Pantothenic acid	5 mg	ND	ND	ND
415	Vitamin B6	1.3 mg	100 mg	ND	ND
418	Vitamin B12	2.4 µg	ND	ND	ND
421	Choline	550 mg	3500 mg	ND	ND
430	Vitamin K	120 µg	ND	ND	ND
435	Folate	400 µg DFE¶	1000 µg DFE	ND	ND
501	Tryptophan	0.005 g/kg**	ND	ND	ND
502	Threonine	0.02 g/kg	ND	ND	ND
503	Isoleucine	0.019 g/kg	ND	ND	ND
504	Leucine	0.042 g/kg	ND	ND	ND

Table A. (Continued)

Nutrient ID	Nutrient name	Minimum intake	Maximum intake	Minimum intake (% of calories)	Maximum intake (% of calories)
505	Lysine	0.038 g/kg	ND	ND	ND
506	Methionine	0.019 g/kg	ND	ND	ND
508	Phenylalanine	0.033 g/kg	ND	ND	ND
510	Valine	0.024 g/kg	ND	ND	ND
512	Histidine	0.014 g/kg	ND	ND	ND
601	Cholesterol	ND	300 mg	ND	ND
605	Trans fat	ND	ND	0	1
606	Saturated fat	ND	ND	0	10
675	Linoleic acid	17 g	ND	5	10
851	α-Linolenic acid	1.6 g	ND	0.6	1.2

The first column lists the IDs of the nutrients in the DRI. The daily recommended energy (calorie) is 3057.39 kcal. For more details, refer to Section 1.2. *ND: not determined; †RAE: retinol activity equivalents; ‡NE: niacin equivalents; ¶DFE: dietary folate equivalents; **g per kg body weight.

Table B. Nutrients used for the calculation of the nutritional similarity between foods

Nutrient ID	Nutrient name	Nutrient ID	Nutrient name
203	Protein	418	Vitamin B12
204	Total lipid	421	Choline
205	Carbohydrate	428	Menaquinone-4
209	Starch	429	Dihydrophylloquinone
210	Sucrose	430	Vitamin K
211	Glucose	435	Folate
212	Fructose	454	Betaine
213	Lactose	501	Tryptophan
214	Maltose	502	Threonine
221	Ethyl alcohol	503	Isoleucine
262	Caffeine	504	Leucine

Table B. (Continued)

Nutrient ID	Nutrient name	Nutrient ID	Nutrient name
263	Theobromine	505	Lysine
269	Total sugars	506	Methionine
287	Galactose	507	Cystine
291	Fiber	508	Phenylalanine
301	Calcium	509	Tyrosine
303	Iron	510	Valine
304	Magnesium	511	Arginine
305	Phosphorus	512	Histidine
306	Potassium	513	Alanine
307	Sodium	514	Aspartic acid
309	Zinc	515	Glutamic acid
312	Copper	516	Glycine
315	Manganese	517	Proline
317	Selenium	518	Serine
320	Vitamin A	521	Hydroxyproline
323	Vitamin E	601	Cholesterol
324	Vitamin D	605	Trans fat
401	Vitamin C	606	Saturated fat
404	Thiamin	645	Monounsaturated fat
405	Riboflavin	646	Polyunsaturated fat
406	Niacin	675	Linoleic acid
410	Pantothenic acid	851	α-Linolenic acid
415	Vitamin B6		

The first column lists the IDs of the nutrients in the DRI. For the calculation of the nutritional similarity between foods, refer to Section 1.3 and Section 3.1.

Table C. Bottleneck nutrients for high nutritional fitness (NF) in each food category

Food category	Nutrient ID	Nutrient name	Regression coefficient	Remark
Protein-rich	421	Choline	0.084	Favorable for NF
	328	Vitamin D	0.080	Favorable for NF
	307	Sodium	0.039	Favorable for NF
	301	Calcium	0.014	Favorable for NF
	204	Total lipid	−0.064	Unfavorable for NF
	601	Cholesterol	−0.037	Unfavorable for NF
	406	Niacin	−0.033	Unfavorable for NF
Fat-rich	675	Linoleic acid	0.489	Favorable for NF
	421	Choline	0.103	Favorable for NF
	301	Calcium	0.025	Favorable for NF
	851	α-Linolenic acid	0.013	Favorable for NF
	315	Manganese	−0.028	Unfavorable for NF
Carbohydrate-rich	205	Carbohydrate	0.362	Favorable for NF
	851	α-Linolenic acid	0.120	Favorable for NF
	421	Choline	0.104	Favorable for NF
	430	Vitamin K	0.015	Favorable for NF
	315	Manganese	−0.021	Unfavorable for NF
	435	Folate	−0.012	Unfavorable for NF
Low-calorie	421	Choline	0.065	Favorable for NF
	851	α-Linolenic acid	0.039	Favorable for NF
	323	Vitamin E	0.038	Favorable for NF
	320	Vitamin A	0.037	Favorable for NF
	301	Calcium	0.014	Favorable for NF
	430	Vitamin K	0.012	Favorable for NF

The second column lists the IDs of the nutrients in the DRI. In the fourth column, the regression coefficient away from zero measures the strength of the corresponding bottleneck nutrient in determining the NFs for the food category in the first column (Section 5.1). The regression coefficient > 0 (< 0) indicates a bottleneck nutrient that is favorable (unfavorable) for NF.

Table D. Synergistic bottleneck pairs of nutrients for high NF

Food category	Nutrient 1	Nutrient 2	Food	Φ_{ij}	*P*	Remark
Protein-rich	Calcium	α-Linolenic acid	Flatfish	8.95	$< 10^{-16}$	F, F
	Carbohydrate	Linoleic acid	Scallop	7.31	2.58×10^{-13}	F, F
	Choline	α-Linolenic acid	Flatfish	6.52	7.19×10^{-11}	F, F
	Vitamin D	Methionine	Ocean perch	5.00	5.67×10^{-7}	F, F
	Carbohydrate	Calcium	Instant nonfat dry milk	4.60	4.26×10^{-6}	F, F
	Carbohydrate	Vitamin D	Roe	4.42	1.00×10^{-5}	F, F
	Selenium	Vitamin A	Bluefin tuna	3.65	2.65×10^{-4}	F, F
	Carbohydrate	Vitamin A	Clam	3.43	5.95×10^{-4}	F, F
	Choline	Phenylalanine	Ocean perch	3.39	6.99×10^{-4}	F, F
	Carbohydrate	Choline	Beef liver	3.28	1.03×10^{-3}	F, F
	Choline	Methionine	Ocean perch	3.07	2.12×10^{-3}	F, F
	Vitamin A	Vitamin B12	Bluefin tuna	2.91	3.61×10^{-3}	F, F
	Vitamin E	Vitamin D	Pacific cod	2.81	5.01×10^{-3}	F, F
	Protein	Choline	Flatfish	2.76	5.81×10^{-3}	F, F
	Vitamin A	Vitamin D	Eel	2.71	6.64×10^{-3}	F, F
	Choline	Linoleic acid	Scallop	2.70	7.02×10^{-3}	F, F
	Protein	Vitamin D	Ocean perch	2.65	7.98×10^{-3}	F, F
	Vitamin B12	Linoleic acid	Flatfish	2.60	9.24×10^{-3}	F, F
	Vitamin A	Vitamin E	Bluefin tuna	2.38	1.75×10^{-2}	F, F
	Carbohydrate	α-Linolenic acid	Scallop	2.36	1.81×10^{-2}	F, F

Table D. (Continued)

Food category	Nutrient 1	Nutrient 2	Food	Φ_{ij}	P	Remark
Protein-rich	Selenium	Vitamin D	Swordfish	2.35	1.86×10^{-2}	F, F
	Vitamin A	α-Linolenic acid	Flatfish	2.35	1.87×10^{-2}	F, F
	Carbohydrate	Selenium	Scallop	2.33	2.00×10^{-2}	F, F
	Carbohydrate	Sodium	Abolone	2.30	2.15×10^{-2}	F, F
	Protein	Carbohydrate	Scallop	2.27	2.32×10^{-2}	F, F
	Vitamin A	Linoleic acid	Sockeye salmon	2.26	2.38×10^{-2}	F, F
	Choline	Leucine	Ocean perch	2.25	2.44×10^{-2}	F, F
	Pantothenic acid	Choline	Roe	2.24	2.51×10^{-2}	F, F
	Sodium	Vitamin D	Flatfish	2.24	2.54×10^{-2}	F, F
	Vitamin A	Methionine	Bluefin tuna	2.18	2.93×10^{-2}	F, F
	Choline	Threonine	Ocean perch	2.15	3.17×10^{-2}	F, F
	Vitamin B12	Cholesterol	Ocean perch	25.38	$< 10^{-16}$	F, U
	Choline	Cholesterol	Ocean perch	22.03	$< 10^{-16}$	F, U
	Linoleic acid	Total lipid	Ocean perch	19.91	$< 10^{-16}$	F, U
	Vitamin E	Total lipid	Ocean perch	16.67	$< 10^{-16}$	F, U
	Vitamin D	Cholesterol	Flatfish	15.50	$< 10^{-16}$	F, U
	Sodium	Manganese	Ocean perch	13.98	$< 10^{-16}$	F, U
	Sodium	Folate	Ocean perch	12.59	$< 10^{-16}$	F, U
	Methionine	Niacin	Flatfish	11.94	$< 10^{-16}$	F, U
	Selenium	Niacin	Flatfish	11.12	$< 10^{-16}$	F, U
	Sodium	Copper	Ocean perch	10.91	$< 10^{-16}$	F, U

Table D. (Continued)

Food category	Nutrient 1	Nutrient 2	Food	Φ_{ij}	*P*	Remark
Protein-rich	Vitamin B12	Folate	Flatfish	10.31	$< 10^{-16}$	F, U
	Vitamin D	Folate	Ocean perch	10.04	$< 10^{-16}$	F, U
	Choline	Folate	Ocean perch	9.69	$< 10^{-16}$	F, U
	Protein	Niacin	Ocean perch	9.49	$< 10^{-16}$	F, U
	Choline	Copper	Ocean perch	8.66	$< 10^{-16}$	F, U
	Choline	Saturated fat	Flatfish	8.07	6.66×10^{-16}	F, U
	Sodium	Cholesterol	Ocean perch	7.47	8.29×10^{-14}	F, U
	Sodium	Iron	Ocean perch	7.00	2.50×10^{-12}	F, U
	Choline	Manganese	Ocean perch	5.48	4.19×10^{-8}	F, U
	Carbohydrate	Saturated fat	Scallop	5.36	8.23×10^{-8}	F, U
	Pantothenic acid	Niacin	Ocean perch	4.85	1.24×10^{-6}	F, U
	α-Linolenic acid	Niacin	Flatfish	4.15	3.26×10^{-5}	F, U
	Sodium	Saturated fat	Atlantic cod	3.99	6.49×10^{-5}	F, U
	Linoleic acid	Niacin	Ocean perch	3.97	7.12×10^{-5}	F, U
	Thiamin	Niacin	Ocean perch	3.87	1.08×10^{-4}	F, U
	Choline	Phosphorus	Ocean perch	3.59	3.34×10^{-4}	F, U
	Vitamin D	Total lipid	Ocean perch	3.53	4.13×10^{-4}	F, U
	Vitamin D	Phosphorus	Pacific jack mackerel	3.53	4.17×10^{-4}	F, U
	Calcium	Total lipid	Instant nonfat dry milk	3.33	8.83×10^{-4}	F, U
	Choline	Vitamin D	Clam	2.83	4.71×10^{-3}	F, U
	Potassium	Niacin	Flatfish	2.71	6.77×10^{-3}	F, U

Table D. (Continued)

Food category	Nutrient 1	Nutrient 2	Food	Φ_{ij}	P	Remark
Protein-rich	α-Linolenic acid	Cholesterol	Sockeye salmon	2.64	8.22×10^{-3}	F, U
	Vitamin K	Saturated fat	Sprouted alfalfa seed	2.54	1.09×10^{-2}	F, U
	Vitamin B12	Niacin	Flatfish	2.53	1.14×10^{-2}	F, U
	Carbohydrate	Manganese	Scallop	2.47	1.35×10^{-2}	F, U
	Vitamin A	Cholesterol	Clam	2.37	1.76×10^{-2}	F, U
	Vitamin A	Saturated fat	Clam	2.27	2.33×10^{-2}	F, U
	α-Linolenic acid	Total lipid	Ocean perch	2.08	3.72×10^{-2}	F, U
	Sodium	Niacin	Ocean perch	2.05	4.00×10^{-2}	F, U
	Phenylalanine	Niacin	Ocean perch	2.01	4.44×10^{-2}	F, U
Fat-rich	Choline	Phosphorus	Almond	22.84	$< 10^{-16}$	F, U
	Choline	Niacin	Almond	21.08	$< 10^{-16}$	F, U
	Choline	Iron	Almond	17.08	$< 10^{-16}$	F, U
	Carbohydrate	Folate	Almond	16.30	$< 10^{-16}$	F, U
	Vitamin E	Niacin	Almond	15.63	$< 10^{-16}$	F, U
	Choline	Folate	Almond	14.11	$< 10^{-16}$	F, U
	Vitamin E	Manganese	Almond	10.57	$< 10^{-16}$	F, U
	Vitamin E	Folate	Almond	10.50	$< 10^{-16}$	F, U
	Vitamin E	Iron	Almond	9.10	$< 10^{-16}$	F, U
	Choline	Manganese	Almond	8.46	$< 10^{-16}$	F, U
	Calcium	Manganese	Almond	7.83	4.77×10^{-15}	F, U
	Carbohydrate	Niacin	Almond	6.75	1.47×10^{-11}	F, U

Table D. (Continued)

Food category	Nutrient 1	Nutrient 2	Food	Φ_{ij}	*P*	Remark
Fat-rich	Calcium	Iron	Almond	6.41	1.46×10^{-10}	F, U
	Calcium	Selenium	Almond	5.31	1.11×10^{-7}	F, U
	Choline	Vitamin C	Almond	5.15	2.65×10^{-7}	F, U
	Vitamin E	Sodium	Almond	4.23	2.29×10^{-5}	F, U
	Protein	Manganese	Pork separable fat	2.61	8.94×10^{-3}	F, U
	Calcium	Vitamin A	Almond	2.39	1.71×10^{-2}	F, U
	Calcium	Niacin	Almond	2.33	1.96×10^{-2}	F, U
	Total lipid	Folate	Almond	22.97	$< 10^{-16}$	U, U
	Folate	Saturated fat	Almond	34.55	$< 10^{-16}$	U, U
	Total lipid	Niacin	Almond	17.38	$< 10^{-16}$	U, U
Carbohydrate-rich	Vitamin A	Vitamin E	Frozen green pea	3.26	1.10×10^{-3}	F, F
	Pantothenic acid	Manganese	Cherimoya	14.45	$< 10^{-16}$	F, U
	Potassium	Manganese	Cherimoya	7.81	5.88×10^{-15}	F, U
	Thiamin	Manganese	Cherimoya	6.26	3.89×10^{-10}	F, U
	Vitamin B6	Manganese	Cherimoya	6.16	7.44×10^{-10}	F, U
	Choline	Phosphorus	Tangerine	4.62	3.80×10^{-6}	F, U
	Vitamin K	Saturated fat	Frozen green pea	4.17	3.00×10^{-5}	F, U
	Choline	Iron	Fordhook lima bean	3.11	1.85×10^{-3}	F, U
	Vitamin K	Manganese	Green kiwifruit	2.88	3.93×10^{-3}	F, U
	Calcium	Folate	Gold kiwifruit	2.72	6.51×10^{-3}	F, U
	Choline	Saturated fat	Frozen green pea	2.64	8.25×10^{-3}	F, U

Table D. (Continued)

Food category	Nutrient 1	Nutrient 2	Food	Φ_{ij}	*P*	Remark
Carbohydrate-rich	Vitamin K	Folate	Dried plum	2.56	1.04×10^{-2}	F, U
	Vitamin A	Niacin	Gold kiwifruit	2.53	1.13×10^{-2}	F, U
	Vitamin E	Total lipid	Tangerine	2.53	1.15×10^{-2}	F, U
	Calcium	Iron	Kumquat	2.36	1.83×10^{-2}	F, U
	Choline	Folate	Gold kiwifruit	2.33	1.96×10^{-2}	F, U
	Choline	Total lipid	Clementine	2.33	1.99×10^{-2}	F, U
	Choline	Manganese	Fordhook lima bean	2.32	2.05×10^{-2}	F, U
	Vitamin K	Phosphorus	Dried plum	2.06	3.93×10^{-2}	F, U
	Vitamin A	Iron	Gold kiwifruit	2.06	3.98×10^{-2}	F, U
Low-calorie	Sodium	Zinc	Beet green	3.28	1.03×10^{-3}	F, F
	Carbohydrate	Sodium	Dried celery flake	2.33	1.99×10^{-2}	F, F
	Carbohydrate	Zinc	Dried parsley	2.62	8.83×10^{-3}	F, F
	Choline	Vitamin D	Swiss chard	10.28	$< 10^{-16}$	F, U
	Choline	Niacin	Fresh basil	7.11	1.12×10^{-12}	F, U
	Calcium	Iron	Watercress	6.93	4.29×10^{-12}	F, U
	Vitamin K	Folate	Swiss chard	6.92	4.51×10^{-12}	F, U
	Calcium	Folate	Swiss chard	6.47	9.79×10^{-11}	F, U
	Calcium	Manganese	Swiss chard	5.52	3.36×10^{-8}	F, U
	Vitamin A	Folate	Swiss chard	5.52	3.47×10^{-8}	F, U
	Zinc	Niacin	Dried parsley	4.86	1.17×10^{-6}	F, U
	Sodium	Total lipid	Beet green	4.59	4.51×10^{-6}	F, U

Table D. (Continued)

Food category	Nutrient 1	Nutrient 2	Food	Φ_{ij}	*P*	Remark
Low-calorie	Vitamin K	Iron	Swiss chard	4.43	9.34×10^{-6}	F, U
	Calcium	Total lipid	Swiss chard	3.72	2.00×10^{-4}	F, U
	Zinc	Iron	Raw zucchini	3.49	4.81×10^{-4}	F, U
	Choline	Phosphorus	Red cabbage	3.31	9.48×10^{-4}	F, U
	Zinc	Vitamin D	Dried parsley	3.23	1.24×10^{-3}	F, U
	Vitamin K	Manganese	Swiss chard	2.98	2.84×10^{-3}	F, U
	Vitamin K	Saturated fat	Swiss chard	2.79	5.21×10^{-3}	F, U
	Vitamin A	Niacin	Red cabbage	2.79	5.33×10^{-3}	F, U
	Choline	α-Linolenic acid	Swiss chard	2.71	6.66×10^{-3}	F, U
	Vitamin E	Iron	Watercress	2.64	8.36×10^{-3}	F, U
	Zinc	Manganese	Raw zucchini	2.61	9.07×10^{-3}	F, U
	Choline	Saturated fat	Watercress	2.50	1.25×10^{-2}	F, U
	Choline	Copper	Red cabbage	2.47	1.34×10^{-2}	F, U
	Choline	Selenium	Red tomato	2.44	1.48×10^{-2}	F, U
	α-Linolenic acid	Folate	Chili powder	2.32	2.06×10^{-2}	F, U
	Calcium	Saturated fat	Broccoli raab	2.01	4.41×10^{-2}	F, U

For each food category, we list the synergistic bottleneck pairs ($\Phi_{ij} > 2.0$) composed of nutrients in Table A. Only a food in which a given pair of nutrients exhibits the strongest synergism (among multiple foods) for high NF is shown in the fourth column. Because Φ_{ij} itself is derived from a two-sided *Z*-test (Section 6.1), its *P* value calculation is straightforward, and the value is shown in the sixth column. In the seventh

column, ‘F’ (‘U’) denotes that the nutrient is ‘favorable’ (‘unfavorable’) for high NF in the food of the fourth column. For example, ‘F, U’ means that a nutrient in the second column is favorable, while the other nutrient in the third column is unfavorable. Only the pairs of nutrients that were assigned to be definitely ‘F’ or ‘U’ appear in this table (Section 6.1).

Table E. Nutrients considered for the construction of the nutrient-nutrient network

Nutrient ID	Nutrient name	Nutrient ID	Nutrient name
203	Protein	320	Vitamin A
204	Total lipid	323	Vitamin E
205	Carbohydrate	328	Vitamin D
209	Starch	401	Vitamin C
210	Sucrose	404	Thiamin
211	Glucose	405	Riboflavin
212	Fructose	406	Niacin
213	Lactose	410	Pantothenic acid
214	Maltose	415	Vitamin B6
263	Theobromine	418	Vitamin B12
269	Total sugar	421	Choline
287	Galactose	428	Menaquinone-4
291	Fiber	429	Dihydrophylloquinone
301	Calcium	430	Vitamin K
303	Iron	435	Folate
304	Magnesium	454	Betaine
305	Phosphorus	601	Cholesterol
306	Potassium	605	Trans fat
307	Sodium	606	Saturated fat
309	Zinc	645	Monounsaturated fat
312	Copper	646	Polyunsaturated fat
315	Manganese	675	Linoleic acid
317	Selenium	851	α-Linolenic acid

The first and third columns list the IDs of the nutrients in the DRI. For more details, see Section 7.2.
